# A Welcome Proposal to Amend the GMO Legislation of the EU

**DOI:** 10.1016/j.tibtech.2018.05.001

**Published:** 2018-11

**Authors:** Dennis Eriksson, Wendy Harwood, Per Hofvander, Huw Jones, Peter Rogowsky, Eva Stöger, Richard G.F. Visser

**Affiliations:** 1 Department of Plant Breeding, Swedish University of Agricultural Sciences, 23053 Alnarp, Sweden; 2 John Innes Centre, Norwich Research Park, Norwich, NR4 7UH, UK; 3 Institute of Biological, Environmental and Rural Sciences (IBERS), Aberystwyth University, Aberystwyth, SY23 3EB, UK; 4 Laboratoire Reproduction et Développement des Plantes, Univ Lyon, ENS de Lyon, UCB Lyon 1, CNRS, INRA, F-69342, Lyon, France; 5 Department of Applied Genetics and Cell Biology, University of Natural Resources and Life Sciences, Vienna, Austria; 6 Plant Breeding, Wageningen University & Research, PO Box 386, 6700 AJ Wageningen, The Netherlands

**Keywords:** new plant breeding techniques, gene editing, GMO, EU Directive

## Abstract

Is the European Union (EU) regulatory framework for genetically modified organisms (GMOs) adequate for emerging techniques, such as genome editing? This has been discussed extensively for more than 10 years. A recent proposal from The Netherlands offers a way to break the deadlock. Here, we discuss how the proposal would affect examples from public plant research.

New plant breeding techniques (NPBTs) developed over the past two decades have provided ample possibilities for efficient and elaborate plant research and trait development. However, diverging stakeholder opinions and politically motivated arguments have left policy-makers in the EU bereft of license to decide on their status in relation to existing regulations. Some EU member states have already been compelled by various requests from seed companies and public research groups to interpret unilaterally the applicability of the EU legislation to certain NPBTs. Therefore, acknowledging the need for progress and political resolution, The Netherlands Ministry of Infrastructure and the Environment recently proposedi to amend the EU Directive 2001/18/EC [1] on the deliberate release into the environment of GMOs.

## The Problem with the Current EU GMO Legislation

Annex 1B of the EU Directive 2001/18/EC lists techniques, such as mutagenesis, resulting in GMOs that are excluded from regulation by the Directive. There are two problems here: the Directive contains no definition of mutagenesis, and the definition of GMO is somewhat ambiguous because it refers to an organism ‘in which the genetic material has been altered in a way that does not occur naturally’. Another complicating factor is that the national translations of the Directive contain wording regarding the definitions of GMO and mutagenesis that may differ in interpretation, and an attempt at harmonization would be desirable. A common interpretation among the scientific community of the GMO definition, shared by us and others [2], is that a certain technique may serve as a trigger for the classification as GMO, but that a certain outcome regarding the product (i.e., the presence of a novel combination of stably inherited recombinant nucleic acids) is also required for an organism to be regulated under Directive 2001/18/EC. However, not all stakeholders agree with this interpretation, hampering the political progress to decide on the regulation of NPBTs.

## A Proposal to Amend the EU GMO Directive

The Dutch proposal does not seek to reconcile the different interpretations of the GMO definition *per se*, but merely recommends a set of criteria to replace the list of techniques in Annex 1B (Box 1). Not all products resulting from the use of NPBTs would be exempted; instead, exemption would be based on the dual condition that: (i) the resulting organism does not contain recombinant nucleic acids; and (ii) no genetic material other than what could be exchanged through traditional breeding methods is introduced. It is not the first proposal of this kind to originate from The Netherlands. In 2006, a team at Wageningen University and Research [3] suggested that cisgenesis, one of the eight NPBTs mentioned in a report requested by the EU [4], should be listed among the exemptions in Annex 1B. The new proposal is also consistent with several reports delivered by The Netherlands Commission on Genetic Modification (COGEM) 5, 6, 7.

Box 1 **The Original Intentions of the EU GMO Legislation**

The Dutch proposal is compatible with the original intentions for GMO regulation in the EU [13]. The 1988 draft for a Council Directive advertises ‘the commitment to update the Directive to technical progress as necessary, given the rapid scientific development’ and declares further that ‘the Commission shall adapt the annexes of this Directive to technical progress by amending new techniques’ [14]. However, this provision was not included in later Directives 1, 15. Nearly 30 years of technical progress is arguably a compelling reason to endow the current Directive with such a mechanism. When the GMO Directive was developed, transgenesis mainly aimed at ectopic expression of foreign genes, whereas techniques such as genome editing or RNA interference were not yet developed. Today, researchers and breeders use these and other new breeding techniques, such as synthetic oligonucleotides or optimized alleles with very high similarity to endogenous sequences, to alter the sequence or expression patterns of endogenous genetic material in more subtle ways. Additionally, horizontal gene transfer occurs commonly in nature, and genotypes of a given species can display more dissimilar genomes than may have been thought previously.

Alt-text: Box 1

## Examples from Public Research Institutes

We, being publicly funded academic researchers, welcome the intentions of the Dutch proposal because we acknowledge that periodically updating the regulatory framework is instrumental in responding to technical progress. Here, we present examples of publicly funded research, from our own or other institutes, resulting in applications that may be affected by the proposal if it were to be adopted into EU legislation.

### Austria

Several research programs have performed targeted genome editing with the CRISPR/Cas9 system. One example is barley in which genomic fragment deletions have been produced in the endo-*N*-acetyl-β-d-glucosaminidase (ENGase) gene [8] to alter glycoprotein modifications in cereal seeds. Additional target gene knockouts in barley and *Nicotiana* species include regulators of endoplasmic reticulum (ER) stress and metabolic pathways to improve the suitability and performance of plants for the production of pharmaceutically and industrially valuable compounds. To the extent that the resulting plants do not contain recombinant nucleic acids, they would not be regulated as GMOs according to the Dutch proposal.

### France

The research project GENIUSii (2012–2019) implements genome editing as a research and breeding tool in crops. Both gene inactivation by random modifications and allele replacement by specific, matrix-based modifications have been achieved at predetermined sites. The traits chosen for proof of concept differ between the nine species under investigation (rice, wheat, maize, tomato, potato, oilseed rape, poplar, apple, and rose) and include disease resistance to viruses and fungi, flowering time, plant architecture, tolerance to salinity, and plant reproduction. These traits promote a more sustainable agriculture by the reduction of pesticides and the mitigation of climate change. Following the Dutch proposal, it is clear that several examples developed in GENIUS would not be subject to the GMO regulation.

### The Netherlands

The Durable Resistance against *P. infestans* (DuRPh) project (2006–2016) aimed to create cisgenic potatoes carrying multiple late blight-resistance genes from crossable wild species. Plants carrying different resistance genes in different varietal backgrounds were created. Among the events obtained via *Agrobacterium* transformation, a few were identified carrying only the resistance genes with regulatory sequences and no other (nonpotato) DNA sequences. Many different events have been field tested over the years and remained unaffected by the disease. Clean events (which are true to type) are ideal cases for market introduction [9], because no other genetic material is introduced other than what can be exchanged through traditional breeding.

### Sweden

Genome editing is applied on potato via the transfection of protoplasts and subsequent regeneration of plants, to produce tubers with an improved starch quality for industrial extraction. CRISPR/Cas9 and double-stranded breaks with nonhomologous end-joining (NHEJ) are utilized to achieve mutations and enzyme function knockout, and successful multiallelic mutations in regenerated potato plants have been demonstrated [10]. The resulting genetic alterations are not of recombinant type and would not be regulated as GMO.

### UK: England

**Figure 1:**
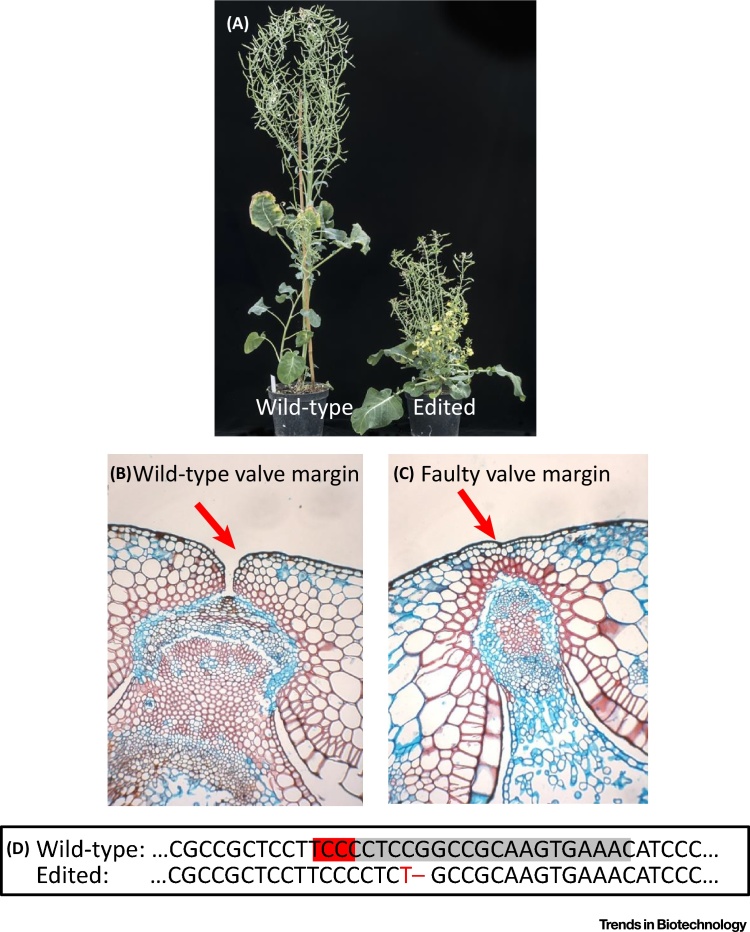
Genome Editing to Reduce Pod Shatter in *Brassica* Crops. (A) A wild-type *Brassica oleracea* plant and a CRISPR/Cas9 mutant showing the expected dwarf phenotype. (B) Section through the pod of a wild-type plant showing the normal valve margin. (C) Section through the pod of a CRISPR mutant plant showing the faulty valve margin. (D) Part of the sequence of the *BolC.GA4.a* gene, showing the target site in the wild-type (gray box with PAM motif in red) and a mutant allele showing the change in red. Wild-type and edited plants in (A) adapted from [11].

Targeted gene knockouts are produced using RNA-guided Cas9 in a range of crops, including barley, wheat, *Brassica oleracea*, *Brassica napus*, potato, and tomato. This technology is valuable for research on abiotic stress tolerance, disease resistance, aspects of plant development, nitrogen use efficiency, and food and feed quality. There are also real opportunities for crop improvement. One example is *B. oleracea*, where CRISPR/Cas9 has been used to produce edited plants with shatter-resistant pods to reduce losses during harvest (Figure 1) [11]. In the cereals, there is the potential to engineer drought tolerance through specific mutations that will affect the regulation of transcription factors involved in the stress response. These mutations would also, with the Dutch proposal, lead to organisms that are not regulated as GMOs.

### UK: Wales

*Lolium perenne*, an important forage and turf grass, has never been domesticated to lose its innate seed-shattering phenotype. This is a major yield constraint for the producers of certified seed, who currently must harvest the seeds before they are fully mature or use other expensive recovery methods. Homologs of shattering genes are identified in *L. perenne* and single base-pair substitutions yield a nonshattering phenotype. Grasses are outbreeding polyploids that are bred as families rather than as individuals, which makes this trait intractable using conventional methods. This breeding bottleneck is being overcome by using CRISPR/Cas9 gene editing to make this conversion in elite *L. perenne* germplasm, without introducing any recombinant nucleic acids.

## Complications and Prospects

Although the resulting plants in many of the examples presented above would have only point mutations or deletions in endogenous genes, or the addition of genetic material from crossable species, both public and private breeders, including in particular small and medium-sized enterprises, would understandably be reluctant to incorporate this material into their breeding populations if it would then be regulated as a GMO. Hence, there is a risk that over-regulation of emerging gene technologies will lead to lost opportunities for improved crops. We are of the opinion that many of the above presented examples may already not be covered by GMO regulations under the current Directive, but the Dutch proposal to amend Annex 1B would provide necessary clarification beyond doubt for all stakeholders.

The proposal is not free from complications. Some stakeholders are reluctant to open up the EU GMO legislation for amendments, because such a process could take many years, during which the plant research and breeding sector would lack a clear vision for commercial applications. Opening up the Directive might also lead to increased regulation of so-called ‘conventional breeding techniques’, which would be detrimental to the entire seed sector. The proposal may be untimely because all attention is presently directed towards a European Court of Justice case on mutagenesis (C528/16), which may be decisive for certain applications of genome editing [12]. However, this court case is limited in scope and will not resolve the issue of NPBTs other than applications of nuclease- or nucleotide-directed mutagenesis. It is also argued that amending Annex 1B is unnecessary since, according to a common interpretation, Directive 2001/18/EC is already sufficiently clear to manage NPBTs. However, more than 10 years of discussions demonstrate that this is not the case. Although it may still suffer from shortcomings, the Dutch proposal is a welcome approach to initiate a dialog at the EU level and may offer the possibility for the research at our institutes, together with many others, to deliver benefits to farmers, the environment, and society.

## Resources

^i^www.rijksoverheid.nl/binaries/rijksoverheid/documenten/kamerstukken/2017/09/13/proposal-for-discussion/proposal-for-discussion.pdf

^ii^www.genius-project.fr/en/
